# Molecular Identification of First Putative Aquaporins in Snails

**DOI:** 10.1007/s00232-014-9629-0

**Published:** 2014-01-21

**Authors:** Joanna R. Pieńkowska, Ewa Kosicka, Małgorzata Wojtkowska, Hanna Kmita, Andrzej Lesicki

**Affiliations:** 1 Department of Cell Biology, Institute of Experimental Biology, Faculty of Biology, Adam Mickiewicz University in Poznan, Umultowska 89, 61-614 Poznań, Poland; 2 Laboratory of Bioenergetics, Institute of Molecular Biology and Biotechnology, Faculty of Biology, Adam Mickiewicz University in Poznan, Umultowska 89, 61-614 Poznań, Poland

**Keywords:** Gastropoda, *Lymnaea stagnalis*, *Catascopia occulta*, *Stagnicola palustris*, Aquaporin

## Abstract

Aquaporins (AQPs), also known as water channel proteins, are members of a large protein family termed Major Intrinsic Proteins (MIP). The mammalian AQPs have been most comprehensively described, while knowledge about AQPs in invertebrates is limited mainly to insects. Not a single AQP protein has been described in snails to date. Consequently, we decided to search for the proteins in gastropod representatives, namely *Lymnaea stagnalis*, *Catascopia occulta*, and *Stagnicola palustris* (Mollusca; Gastropoda; Pulmonata; Lymnaeidae). Using the molecular approach, we identified *L. stagnalis, C. occulta,* *and S. palustris* open reading frames (ORFs) showing homology to AQP genes available in GenBank database, and characterized the encoded proteins, referred to as LsAQP1, CoAQP1, and SpAQP1, respectively. The putative snail aquaporins contain 299 amino acids, have a molecular mass of about 32 kDa, display the general AQP topology and three-dimensional structure congruent with orthodox AQPs, i.e., water-specific ones. Due to high levels of similarity in their characteristics, LsAQP1 was chosen for further studies, as the obtained results were supposed to be applicable for CoAQP1 and SpAQP1. Expression analysis revealed the presence of *LsAQP1* transcript in the digestive tract, the cerebral ganglia, the kidney, the reproductive system, and the foot, suggesting that LsAQP1 as well as CoAQP1 and SpAQP1 are ubiquitous proteins and may play important roles in many essential water transport processes. The role appears to be confirmed by results of the yeast growth complementation assay pointing at functionality of LsAQP1. Thus, the obtained results support the AQP expression in gastropod tissues for the first time.

**Electronic supplementary material:**

The online version of this article (doi:10.1007/s00232-014-9629-0) contains supplementary material, which is available to authorized users.

## Introduction

The water channel proteins aquaporins (AQPs) belong to the Major Intrinsic Protein superfamily (MIP) that presently includes over 6000 members. The channels facilitate rapid movement of water across cell membranes and have been found in representatives of Bacteria, Archaea, and Eukarya, although the best characterized are mammalian AQPs, particularly human AQPs. Up to now, 13 distinct AQPs named from AQP0 to AQP12 have been identified in humans (Ishibashi et al. 2009). Moreover, all described vertebrate AQPs have their orthologs in humans. Basically, according to the type of transported molecule, AQPs are divided into two subfamilies: orthodox AQPs—exclusively water selective, and aquaglyceroporins (AQGPs)—also permeable to water and other uncharged molecules like glycerol, urea, CO_2_, NO, H_2_O_2_, NH_3_, As(OH)_3_, Sb(OH)_3_, Si(OH)_4_, and Bo (Agre et al. 2002; Bienert et al. 2007; Endeward et al. 2006; Herrera et al. 2006; Herrera and Garvin 2011; Holm et al. 2005; Ma et al. 2006; Meng et al. 2004; Saparov et al. 2007; Tanaka et al. 2008; Verkman and Mitra 2000). Transport through AQPs is bidirectional and mainly dependent upon the concentration gradient of the transported molecule across the membrane. The type of transported molecule depends on the structure of a selectivity filter, also known as aromatic/arginine (ar/R) region, formed by four amino acid residues that interact with passing molecules (Beitz et al. 2006).

Recently, a new system of AQP classification has been proposed that includes four AQP subfamilies in both plants and animals, with 13 ortholog clusters defined (Soto et al. 2012). The only criterion for separating subfamilies is the level of amino acid sequence identity, not the kind of transported molecule. At this basis, it has been suggested that plant and animal AQPs derive from four ancestral AQP subfamilies: A (PIP-like and AQP1-like), B (TIP-like and AQP8-like), C (NIP-like and AQP3-like), and D (SIP-like and AQP11-like). Thus, according to the proposition, animal AQPs should be classified as follows: AQP1-like (includes AQP0, 1, 2, 4, 5, and 6 ortholog clusters containing typical orthodox AQPs), AQP3-like (with AQP3, 7, 9, and 10 ortholog clusters representing AQGPs), AQP8-like (contains only AQP8 ortholog cluster, orthodox AQP), and AQP11-like (with AQP11, and 12 ortholog clusters, unorthodox AQPs).

All AQPs share an overall topology and protein structure in the cell membrane (Sui et al. 2001). The functional channels are formed by four AQP monomers (Agre et al. 2002). Each monomer contains six transmembrane domains (I–VI) and five loops (A–E). Both the N- and C-terminal ends are located in the cytosol. The full-length sequence of AQP monomer is arranged as tandem repeats indicating a half-sized gene duplication event during AQP gene origination. One of the most characteristic features of the AQP structure is the two conservative NPA motives (Asn-Pro-Ala), one in each repeat although they can contain some modifications, e.g., Asn-Pro-Ser or Asn-Pro-Val (Gupta et al. 2012; Ishibashi 2006).

Since the discovery of AQPs, studies have been concentrated mainly on the proteins derived from vertebrate tissues. Knowledge of invertebrate AQPs is much more limited (Campbell et al. 2008; Tomkowiak and Pienkowska 2010), as they are known mainly in insects (Spring et al. 2009). In NCBI databases, a few reports have been annotated and they concern identification of AQPs in nematodes, arachnids, crustaceans, tardigrades, flatworms, and segmented worms. In the case of molluscs, only a few sequences of bivalve AQPs and a few ESTs annotated as fragments of putative gastropod AQPs are available. This lack of data is surprising because of the large number of molluscan species and their diverse physiology. Molluscs including snails represent one-fifth of all world animal species, and their habitat is usually strictly connected with water. Thus, an explanation of how they cope with osmoregulation or desiccation in the context of the role(s) of AQPs would be highly desirable. We have therefore chosen three snail species belonging to Lymnaeidae family, i.e., freshwater pulmonate gastropods, namely *Lymnaea stagnalis*, *Stagnicola palustris,* and *Catascopia occulta* (named also *Stagnicola terebra*—see: Vinarski 2003; Vinarski and Glöer 2008; Welter-Schultes 2012). *Lymnaea stagnalis* and *S. palustris* are common in Poland, whereas *C. occulta* is very rare (Rybska et al. 2008). Moreover, the two former species are regarded as representatives of more closely related genera as compared to the *Catascopia* genus (Meier-Brook and Bargues 2002), recently synonymised with *Ladislavella* (Vinarski 2012). *Lymnaea stagnalis,* the only species of freshwater snail with relatively well-characterized transcriptome, is treated almost as a model organism. For this reason, we selected it for AQP identification. The other two lymnaeid species were used to analyse AQPs in different species representing the same family.

Here, we have described the first successful cDNA cloning and consecutive molecular characterization of putative gastropod AQPs. The open reading frames identified for *L. stagnalis*, *C. occulta*, and *S. palustris* were termed *LsAQP1, CoAQP1,* and *SpAQP1,* respectively. Their translated sequence conservation as well as predicted topology and structure indicate that LsAQP1, CoAQP1, and SpAQP1 appear to be orthodox AQPs. Moreover, we observed a high degree of similarity among LsAQP1, CoAQP1, and SpAQP1 and their similarity to vertebrate AQP4, which is also reported for all invertebrate orthodox AQPs identified till now. Finally, an expression pattern of LsAQP1, regarded as a representative of CoAQP1 and SpAQP1, analysed at the level of RNA revealed its presence in a variety of tissues and organs. In addition, the yeast growth complementation assay confirmed functionality of LsAQP1. In summary, the data provide the first and important information concerning gastropod AQPs. Interestingly, LsAQP1, CoAQP1, and SpAQP1 encoding nucleotide sequences appear to reflect relationships between the chosen species based on morphological criteria.

## Materials and Methods

### Snail Material

Adult *L. stagnalis* specimens were collected in 2011 in a pond near the Warta River in Puszczykowo, Wielkopolska Region, Poland (52°16′12.52″N; 16°52′27.67″ E). *Catascopia occulta* (= *Ladislavella terebra*) specimens were collected in 2011 in a drainage ditch near Gorzykowo, Wielkopolska Region, Poland (52°24′30.57″ N; 17°42′57.34″ E). Specimens of the *Stagnicola palustris* were taken in 2011 and 2012 from mid-field pond water in Poznan, Wielkopolska Region, Poland (52°28′09.30″ N; 16°55′45.02″ E). Species affiliation was determined on the basis of shell morphology and anatomy of reproductive organs (Jackiewicz 1993, 1998). Selected organs and tissues were carefully dissected (after the Jackiewicz 1998 anatomical schemes), placed in solution protecting RNA degradation (RNA*later* ^®^ *Sigma*-*Aldrich*), and frozen at −80 °C.

### cDNA Cloning and Sequencing

A fragment of a snail foot was dissected, minced, and gently homogenized in a hand-held glass homogenizer in ice-cold TRI-Reagent® (*Sigma*-*Aldrich*). RNA was extracted following manufacturer’s protocol (*Sigma*-*Aldrich*). RNA quantity and quality were estimated by spectrophotometric absorption at 260 nm in a NanoDrop® Spectrophotometer ND-1000 (*Thermo Fisher Scientific*). To generate 3′ end partial cDNA clones, 0.5 μg of total RNA was reverse transcribed using AMV native reverse transcriptase (*EURx*) and Q_T_ primer that consists of 17 nucleotides of oligo (dT) followed by a unique 35 base oligonucleotide sequence necessary for the following RACE procedure (Rapid Amplification of cDNA Ends) (Frohman 1993).

**Fig. 1:**
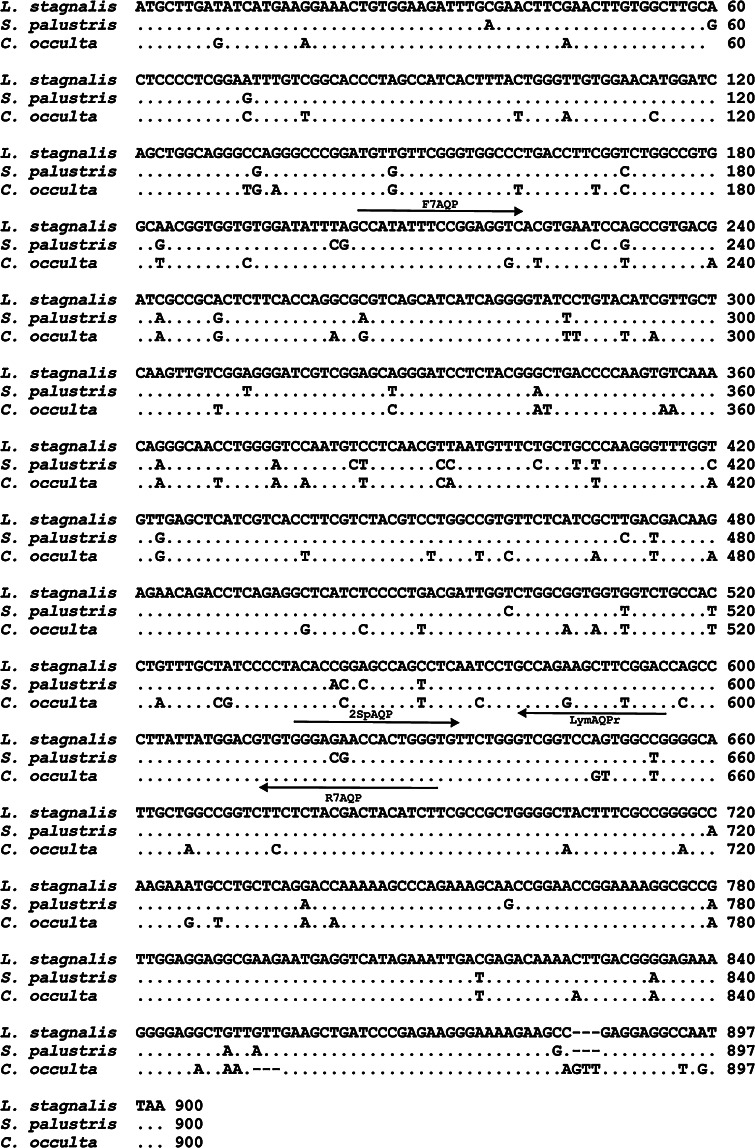
The nucleotide alignment of open reading frames (ORFs) of *LsAQP1*, *CoAQP1*, and *SpAQP1*. *Lines* and *arrows* indicate the placement and directions of the specific primers used in this studies. The polymorphic sites are indicated by *letters*

In the second stage, 30 cycles of PCR were performed: at 94 °C for 30 s; at 55 °C for 30 s; and at 72 °C for 1 min with cDNA as a template and F7AQP and R7AQP primers. The primers were designed on the basis of the EST sequence no. CN810625 obtained in the project analysing *L. stagnalis* cerebral ganglia transcriptome (Feng et al. 2009). All primers applied in the study are presented in Table 1 and indicated in Fig. 1. The amplified fragments were subjected to 1 % agarose gel electrophoresis, purified from reaction mixture by the EZ-10 Spin Column PCR products purification Kit (*Lab Empire*), and sequenced in a 3130xl Genetic Analyser (*ABI Hitachi*). The obtained products were used to design new gene-specific primers needed for following experiments.

**Table 1:** The primers used for the cloning of cDNA of putative lymnaeid aquaporins

Primer	Sequence (from 5′ to 3′)
F7AQP	AGCCATATTTCCGGAGGTC
2SpAQP	ACGTGTGGGAGAACCACTG
R7AQP	ACCCAGTGGTTCTCCCACAC
LymAQPr	CCGAAGCTTCTGGCAGG

In the subsequent step, the RACE technique was used to clone the full-length cDNAs. The 3′ RACE was performed according to the Frohman’s procedure (Frohman 1993), whereas the 5′ RACE was carried out using the 5′/3′ RACE Kit, 2nd Generation (*Roche*). For 3′ RACE-PCR F7AQP and 2SpAQP, gene-specific primers were used; R7AQP and LymAQPr primers were applied to 5′ RACE-PCRs. 2SpAQP and LymAQPr primers were designed on the basis of partial AQP cDNA fragments obtained earlier by PCR, as described above. The PCR products were separated by electrophoresis in 1 % agarose gel, then cut out from the gel, and purified using a MiniElute Gel Extraction Kit (*QIAGEN*). The purified PCR products were either directly sequenced or ligated into pGEMTeasy™ vector (*Promega*) and after transformation of *Escherichia coli* DH5α competent cells (*Invitrogen*), sequenced (Applied Biosystems Hitachi 3130xl Genetic Analyser). The sequences, named *LsAQP1, CoAQP1,* and *SpAQP1*, were deposited in NCBI GenBank as KF157952-KF157954, respectively.

### RT-PCR Analysis of AQP Transcripts in Different Tissues and Organs

For comparative analysis of the gene expression of *L. stagnalis*, putative AQP (LsAQP1) expressed in the digestive tract, the cerebral ganglia, the kidney and the reproductive system, total RNA (0.5 μg) from each part of snail body was reverse- transcribed. The synthesized first strand cDNA was then amplified by PCR using the F7AQP and R7AQP primers, as described above (in cDNA cloning and sequencing).

The RT-PCR product had a length of 430 bp. Simultaneously, *L. stagnalis* actin cDNA (GenBank: KF157955) was amplified as an internal control by RT-PCR using primer set: actup (5′-ATGGTNGGNATGGGNCARAAR-3′) and actdown (5′-DATCCACATYTGYTGRAANGT-3′) (Master 1993). The amplified fragments were visualized by 1 % agarose gel electrophoresis.

### Sequence Analysis

The full-length cDNA was assembled using BioEdit v. 7.1.3.0 software (Hall 1999). Homology analyses of both nucleic acid and deduced amino acid sequences were performed using the BLAST algorithms provided by the website of the National Center for Biotechnology Information (http://www.ncbi.nlm.nih.gov/blast/Blast.cgi) (Altschul et al. 1990).

### Structural Predictions

Hydropathy analyses (Kyte and Doolittle 1982) used to predict transmembrane domains were carried out with the ProtScale program provided by the ExPASy Bioinformatics Resource Portal (http://web.expasy.org/cgi-bin/protscale/protscale.pl). The NetPhos 2.0 and NetPhosK 1.0 programs (Blom et al. 1999; Blom et al. 2004), delivered by ExPASy Proteomic Tools (http://expasy.org/tools), were used to determine phosphorylation sites and kinases. Predictions of 3D structures were carried out using the GeneSilico Metaserver (https://genesilico.pl/meta2) (Kurowski and Bujnicki 2003) and analysed using the PyMOL Molecular Graphics System (version 1.1). Human AQP4 (PDB ID: 3GD8) was used as a template for modeling analyses. The quality of the protein model was established using the ProQ—Protein Quality Predictor (http://www.sbc.su.se/~bjornw/ProQ/ProQ.html).

### Phylogenetic Analysis

Representatives of the amino acid AQP sequences were retrieved from GenBank and aligned with the sequences of gastropod proteins using CLUSTALW (Thompson et al. 1994), followed by manual refinement. Phylogenetic analysis was conducted using MEGA (version 4) (Tamura et al. 2007) based on the Neighbor-Joining method with the amino acid substitution model (Saitou and Nei 1987). The data were bootstrapped for 1000 replicates (Felsenstein 1985).

### Yeast Functional Complementation Assay

The functionality of LsAQP1 was tested using the yeast *Saccharomyces cerevisiae* strain YLL043W (MAT a; his3Δ1; leu2Δ0; met15Δ0; ura3Δ0; YLL043w::kanMX4) from the EUROSCARF strain collection (Institute for Microbiology, Johann Wolfgang Goethe University, Frankfurt, Germany). The YLL043W strain (hereafter referred to as Δ*fps1*) is devoid of *FPS1* gene encoding aquaglyceroporin and possess two nonfunctional orthodox AQPs (Ahmadpour et al. 2013). The ORF of LsAQP1 was flanked with EcoRV and MluI restriction sites due to PCR, and was inserted into the plasmid pYX142 (*Novagen*), that contains the *LEU* gene as a selection marker. The presence of LsAQP1 encoding sequence in pYX142 was confirmed by PCR and sequencing, and the construct was used to transform Δ*fps1* yeast cells by electroporation. The obtained transformants were selected on leucine-depleted selective medium containing glucose as the carbon source. Next, single colonies were chosen for the growth complementation assay. First, they were grown overnight at 28 °C on liquid selective medium without leucine. Then, the cultures were diluted to OD of 0.5 (λ = 550 nm) with the selective medium. Functionality of LsAQP1 was evaluated under hyperosmotic stress, induced by supplementing the media with 1 M NaCl. The cell cultures were spotted on the solid media without leucine in 10× dilution series. The plates were incubated for 4 days at 28 °C. The differences in the growth phenotype between the isogenic wild type (BY4741), Δ*fps1* mutant, and Δ*fps1* + LsAQP1 strains were observed. The plates were digitalized using G:Box Chemi-XR5 GENE*Sys* (*Syngene)*.

## Results

### Analysis of the Identified Snail Putative AQP Nucleotide Sequences and of Encoded Proteins

The aim of this study was to identify AQPs in freshwater snails from the Lymnaeidae family, which are representatives of the Gastropoda class from the Mollusca phylum. We began our research by searching the publicly available EST sequences of a normalized *L. stagnalis* central nervous system (CNS) cDNA library (Feng et al. 2009). We established that this library include sequences that after rewriting to amino acid sequences encoded a protein that display a similarity to the MIP family. Compilation of the EST sequences, which were selected on the basis of their similarity to AQP encoding sequences, yielded the full-length cDNA encoding putative AQP. This initial analysis provided the basis for the cloning of this cDNA and in silico analysis of the encoded protein, which we called LsAQP1. We designed AQP-specific PCR primers on the basis of the CN810625 EST nucleotide sequence (Feng et al. 2009) to amplify cDNAs coding for putative AQPs of *L. stagnalis* and also two other lymnaeids: *C. occulta* and *S. palustris*. A snail foot was the source of cDNA. The F7AQP and R7AQP primer set (Table 1; Fig. 1) produced fragments of 430 bp in the case of all studied species. Based on these partial sequences, using the RACE technique, we proved the correctness of the ESTs assemblage in the case of *LsAQP1* and also obtained the completed putative AQP cDNA sequences for *C. occulta,* and *S. palustris,* which we termed *CoAQP1* and *SpAQP1*, respectively.

The entire identified open reading frames (ORFs) that encoded LsAQP1, CoAQP1, and SpAQP1 were of equal length of 897 bp. A comparison of these sequences showed the presence of 108 polymorphic sites and 6 indels (Fig. 1). The *LsAQP1* nucleotide sequence differed from *SpAQP1* in 51 sites (it gives 94.3 % of the nucleotide sequence identity), and from *CoAQP1* in up to 89 sites (90 % of the nucleotide sequence identity). There is one triplet deleted in position 853–855 and one triplet inserted in position 883–885 in the *CoAQP1* sequence when compared to the two other snail ORFs. The differences in the nucleotide sequences reflect the degree of relationship between examined species based on morphological, anatomical, and molecular studies (Bargues et al. 2001, 2003; Jackiewicz 1993, 1998; Meier-Brook and Bargues 2002; Rybska et al. 2008). The obtained differences confirmed that *L. stagnalis* and *S. palustris* are more closely related species when compared to *C. occulta*. As shown in Fig. 1a and b, the observed differences in nucleotide sequence occured mainly in the third codon positions and did not result in the amino acid change.

**Fig. 2:**
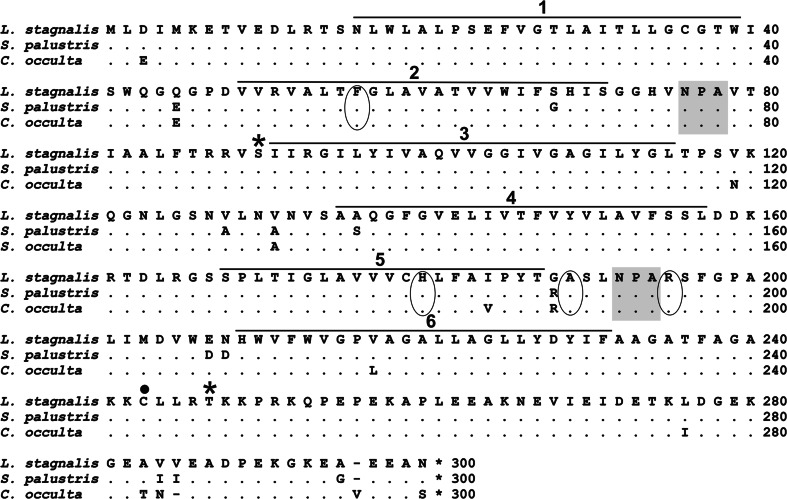
Comparison of deduced amino acid sequences of LsAQP1, CoAQP1, and SpAQP1. Putative transmembrane segments are overlined and numbered from 1 to 6. Two conserved NPA motives, selectivity filters known also as ar/R regions, putative phosphorylation sites, and cysteines—potential Hg^2+^—binding sites are indicated by *shading in gray*, *circles*, *asterisks*, and *dots*, respectively

The predicted proteins contained 299 amino acids, and their estimated molecular weights were 31.9 kDa for both LsAQP1 and SpAQP1, and 32 kDa for CoAQP1. The amino acid sequences of LsAQP1 and SpAQP1 differed in 11 places. LsAQP1 and CoAQP1 have different amino acid residues in 10 positions. In addition, within the CoAQP1 polypeptide sequence there was one amino acid residue lacking in position 285 and one added in position 295 (Val) (Fig. 2).

**Fig. 3:**
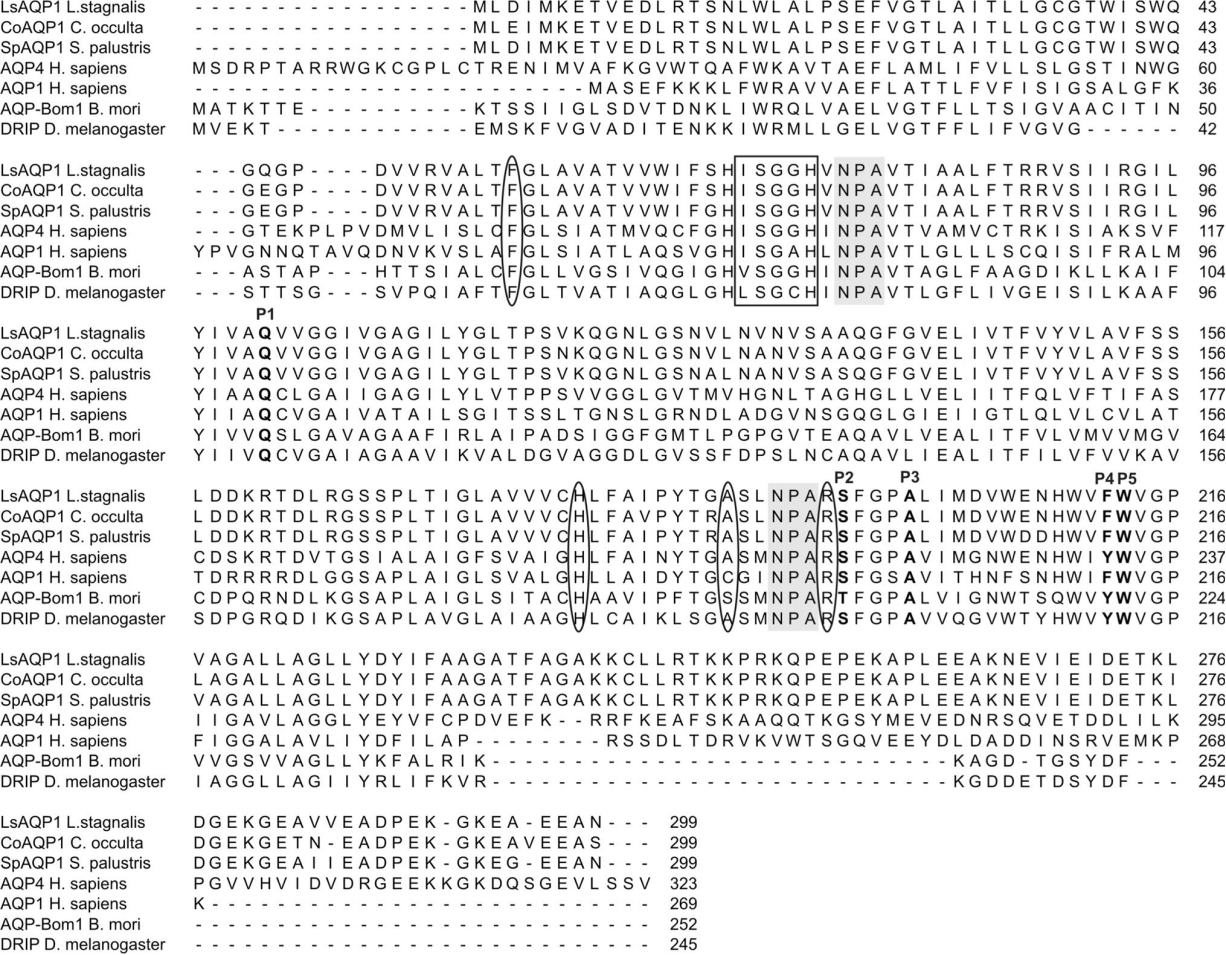
Multiple alignment of predicted amino acid sequences of LsAQP1, CoAQP1, and SpAQP1 and four proteins that have been functionally characterized as orthodox aquaporins: AQP4 from *Homo sapiens* (NP_001641.1), AQP1 from *Homo sapiens* (NP_932766), AQP-Bom1 from *Bombyx mori* (NP_001036919), and DRIP from *Drosophila melanogaster* (NP_523697). Two NPA motives are shaded in *gray*. The amino acids of the selectivity filter (ar/R region) are indicated by *ovals*. The positions P1-P5 and ISGGH sequence, typically conserved in orthodox AQPs, are described by *letters in bold*, and are in a *box*, respectively

BLAST analysis of the amino acid sequences of LsAQP1, CoAQP1, and SpAQP1 revealed their similarity to aquaporins classified as AQP1-like ones. Moreover, as shown in Fig. 3, we compared LsAQP1, CoAQP1, and SpAQP1 with four functionally characterized orthodox AQPs of this subfamily: *Homo sapiens* AQP4 (Hasegawa et al. 1994), *H. sapiens* AQP1 (Preston et al. 1992), *Bombyx mori* AQP-Bom1 (Kataoka et al. 2009), and *Drosophila melanogaster* DRIP (Kaufmann et al. 2005). The amino acid sequence identity among LsAQP1, CoAQP1, and SpAQP1 is about 96 %, whereas the identity between the proteins and the listed above orthodox AQPs was significantly lower. The highest level was achieved in the case of the human AQP4 (about 32 %), and therefore, the analysis of the amino acid sequences and the tree-dimensional structure of the putative lymnaeid AQPs was carried out using the human AQP4 as reference protein.

The sequence analyses of polypeptides encoded by *LsAQP1*, *CoAQP1*, and *SpAQP1* revealed the presence of conserved motives present in all AQPs. These were two Asn-Pro-Ala motives (NPA motives) found in positions 76-78 and 192-194 as well as a selectivity filter, also known as the ar/R region. As mentioned in Introduction, the structure of the ar/R region determined the permeability of AQP channel and, in consequence, is used to classify AQPs as the orthodox AQPs or AQGPs. The ar/R regions of lymnaeid putative aquaporins are formed by four amino acids, namely Phe56, His180, Ala189, and Arg195 (Fig. 2). The same amino acid residues form the selectivity filter of human AQP4 classified within AQP1-like subfamily (Fig. 3). In particular, the presence of histidine within the ar/R region suggested that the studied proteins displayed features of orthodox AQPs. We also identified the ISGGH sequence, located upstream of the first NPA motif, in the positions 70–74 in all putative snail AQPs. This sequence has been found in many orthodox AQPs. In addition, we analysed five positions in polypeptide chain (P1–P5), that are occupied by different amino acids in orthodox AQPs and in AQGPs (Froger et al. 1998). LsAQP1, CoAQP1, and SpAQP1 proteins contained the amino acid residues characteristic to orthodox AQPs in positions P1–P5 (Fig. 3).

As a result of amino acid sequences analysis two potential phosphorylation sites were found in the studied snail proteins; the first one for Ca^2+^/calmodulin-dependent protein kinase II (CaMKII) at Ser90 and the second one for protein kinase C (PKC) at Thr247 (Fig. 2). It has been proven that Cys253 of mammalian AQP4 is a target residue that responds to mercury (Yukutake and Yasui 2010). Thus, cystein in position 243 is supposed to be a mercury-sensitive residue in all three analysed polypeptides (Fig. 2). We did not find any potential site for glycosylation.

**Fig. 4:**
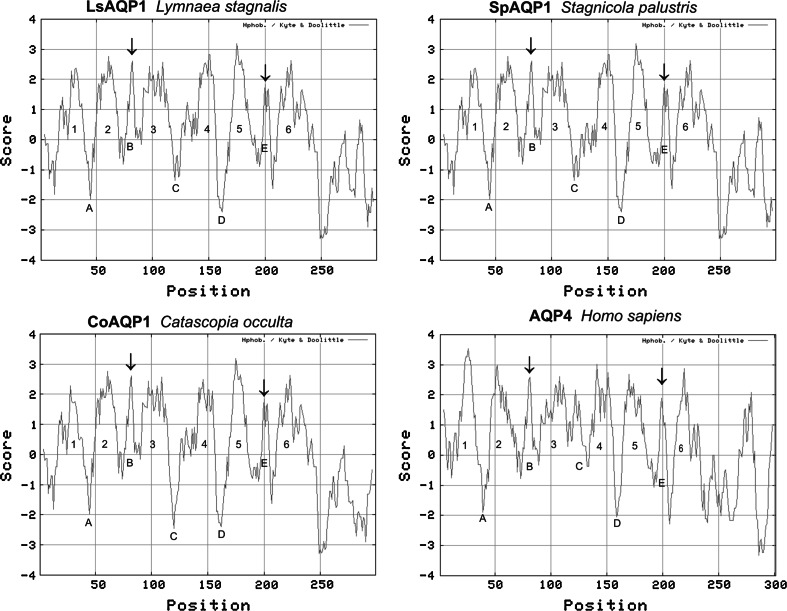
Hydropathy plots of LsAQP1, CoAQP1, and SpAQP1 and orthodox human aquaporin AQP4 (NP_001641.1) (Kyte and Doolittle method with a window width of seven amino acids). Putative transmembrane domains, connecting loops, and NPA motives are shown by the numbers 1–6, letters A–E, and *arrowheads*, respectively

Hydrophaty plots obtained for the deduced amino acid sequences of LsAQP1, CoAQP1, and SpAQP1 using ProtScale program suggested the presence of six transmembrane regions and five loops in the secondary structure of the proteins (Fig. 4). These elements are characteristic features of all AQPs. A similar result was obtained with human AQP4 applied as a control during the same analysis. Therefore, the suggestion that LsAQP1, CoAQP1, and SpAQP1 are members of AQPs is reasonable.

### Three-Dimensional Structure Analysis of LsAQP1, CoAQP1, and SpAQP1

**Fig. 5:**
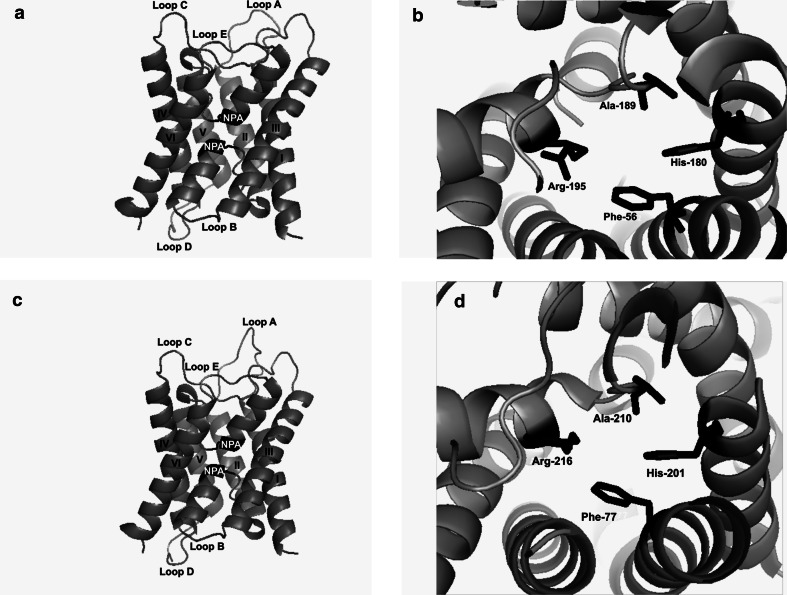
Three-dimensional structure homology model of LsAQP1. The crystal structure of *Homo sapiens* AQP4 (PDB ID: 3GD8) was applied as a template. **a** Three-dimensional homology model of LsAQP1 presented parallel to the plane of the membrane. Two NPA motives are shown in *red/black*. The transmembrane domains and loops are indicated by numbers and letters, respectively. **b** Amino acid residues building the putative selectivity filter known also as ar/R region (Phe56, His180, Ala189, and Arg195) of LsAQP1 are presented in *blue/black*. **c** Three-dimensional structure of human AQP4 (PDB ID: 3GD8) presented in the same orientation as LsAQP1. Description of the picture is analogous to the above. **d** The structure of the human AQP4 selectivity filter with amino acids building this region (Phe77, His201, Ala210, and Arg216) shown in *blue/black* (Color figure online)

Due to a high level of amino acid sequence identity between LsAQP1, CoAQP1, and SpAQP1, LsAQP1 was selected to present results of three-dimensional structure analysis. The results obtained for LsAQP1 are also valid for CoAQP1 and SpAQP1 (Supplementary file 1, Supplementary Material online). A three-dimensional (3D) homology model of LsAQP1 was generated using the GeneSilico Metaserver (Kurowski and Bujnicki 2003) (Fig. 5a). The program indicated the crystallography coordinates of human orthodox AQP4 (PDB ID:3GD8) as the best structural template for LsAQP1, CoAQP1, and SpAQP1 analysis. The quality of the protein model was established using a ProQ—Protein Quality Predictor. The value of LGscore was 4.950, and the value of MaxSub was 0.530. This indicated a very good quality of the model (Wallner and Elofsson 2005). In addition, we compared the obtained 3D model of LsAQP1 to the crystal structure of human AQP4 already existing in the PDB database (Fig. 5c). Six transmembrane domains and two additional membrane embedded α-helices containing two NPA motives were found both in LsAQP1 and in the human AQP4 (Fig. 5a, c). The applied analysis also revealed the presence in LsAQP1 of a structure identical to selectivity filter (ar/R region) of the human AQP4 and built by the same amino acids (Fig. 5b, d). The involved phenylalanine, histidine, alanine, and arginine residues were localized in both proteins in very close positions. For example, the histidine residue identified in position 201 of AQP4 is an important determinant of orthodox AQP. Within the LsAQP1 3D structure, we observed the analogous histidine residue located in 180 position. The compatibility of 3D homology models of LsAQP1 and human AQP4 indicated that they had a similar structure. Thus, it may suggest that LsAQP1 belongs to the orthodox AQPs. The same can be concluded for CoAQP1 and SpAQP1.

### Homology and Phylogenetic Analysis of LsAQP1, CoAQP1, and SpAQP1

**Fig. 6:**
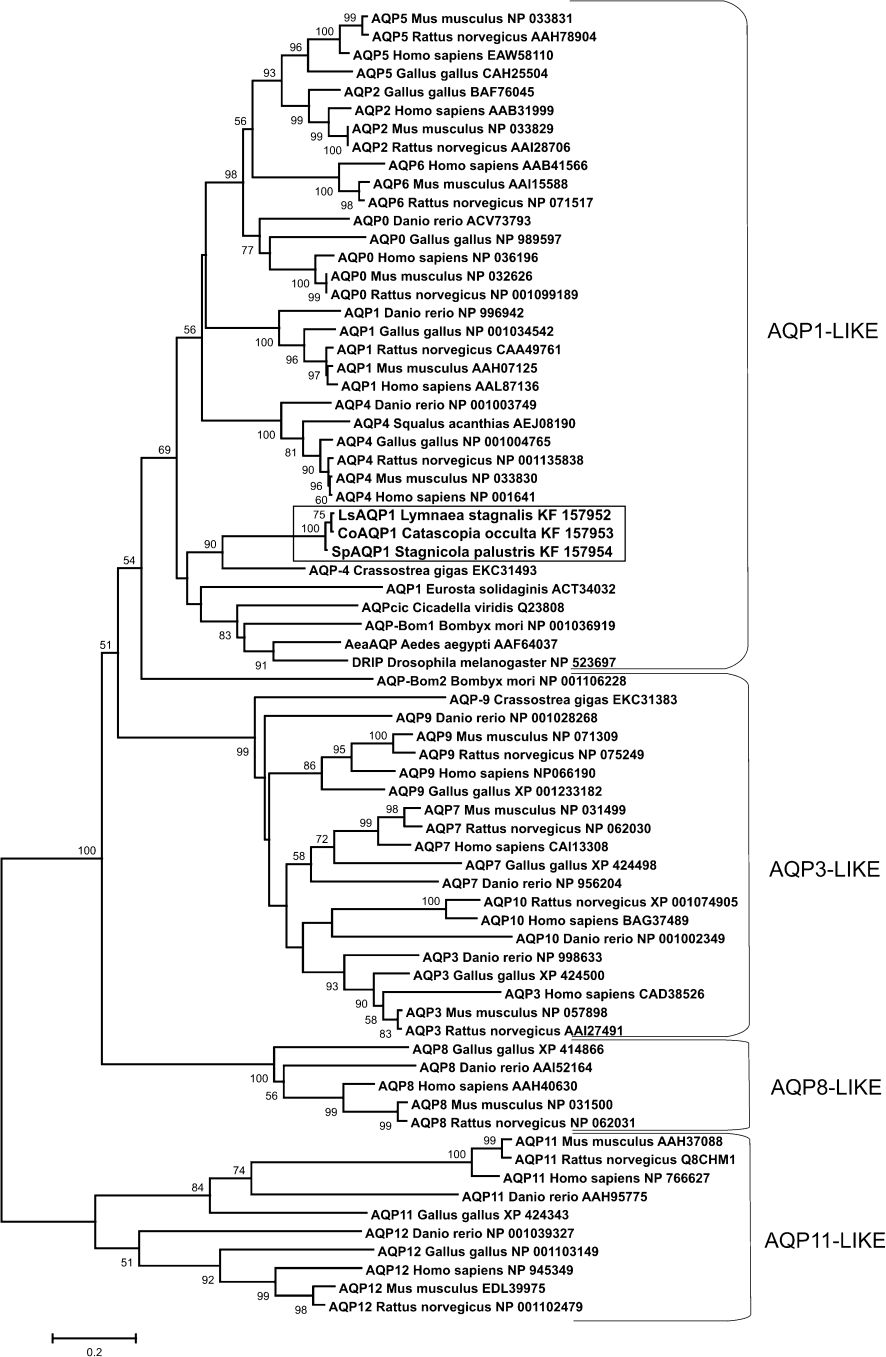
Phylogenetic position and relationships of LsAQP1, CoAQP1, SpAQP1, and selected invertebrate aquaporins within the aquaporin family. The unrooted tree was constructed using the NJ method and tested by bootstrap analysis using 500 replicates with the MEGA program (only bootstrap values >50 are shown). The bar scale indicates 0.2 % amino acid substitutions. The sequences are described by the commonly used acronym, followed by the species’ full name and the accession number. AQP subfamilies names (e.g., AQP1-like) are found on right. Lymnaeid proteins are in *box*

We performed homology and phylogenetic analyses because sequence and structure analysis of LsAQP1, CoAQP1, and SpAQP1 strongly suggested that they belonged to orthodox APQs (AQP1-like subfamily). A multiple sequence alignment (CLUSTALW) was applied to compare LsAQP1, CoAQP1, and SpAQP1 with orthodox AQP sequences that have been thoroughly described for some invertebrates in original publications or with sequences deposited only in the GenBank database. We also made further comparisons of this small invertebrate AQP group with other representatives of AQPs and AQGPs identified both in vertebrates and invertebrates. The results of multiple sequence alignment correlated well with relationships that occurred in the constructed phylogenetic tree (Fig. 6). The tree revealed LsAQP1, CoAQP1, and SpAQP1 association with the majority of invertebrate AQPs within orthodox AQP1-like subfamily (Soto et al. 2012). A bivalvian protein described in the GenBank as AQP4 *Crassostrea gigas* (Zhang et al. 2012) grouped together with LsAQP1, CoAQP1, and SpAQP1. Meanwhile, two other invertebrate proteins, AQP9 from *C. gigas* and AQP-Bom2 from *B. mori*, were localized within the AQP3-like subfamily containing well-known mammalian AQGPs (Soto et al. 2012). Thus, the placement of LsAQP1, CoAQP1, and SpAQP1 in a common branch with well characterized orthodox AQPs seems to be a good indication for assigning the snail AQPs to orthodox AQPs transporting mainly water.

### Expression and Functional Studies of LsAQP1

**Fig. 7:**
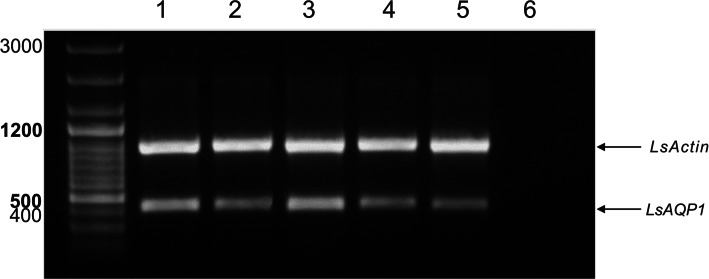
Analysis of the expression of *LsAQP1* at the mRNA level in different organs and tissues. Lines 1 cerebral ganglia; 2 kidney; 3 foot; 4 reproductive system; 5 digestive tract; 6 control reaction without the template. A sample of 0.5 μg of total RNA isolated from the listed above organs and tissues were used as templates for RT-PCR analysis. The products of amplification of *LsAQP1* cDNA fragments (430 bp) and *LsActin* cDNA fragments (941 bp) were analysed using 1 % agarose gel electrophoresis

An expression pattern of *LsAQP1* was established using RT-PCR. The F7AQP and R7AQP primers flanking the cDNA region localized between sequences encoding two NPA motives were used (Table 1; Fig. 1). In the control experiment, degenerate, universal actin-specific primers (Master 1993) were applied to amplify cDNA fragment encoding *L. stagnalis* actin (*LsActin)*. Total RNA isolated from the digestive tract, the cerebral ganglia, the kidney, the reproductive system, and the foot of *L. stagnalis* were used as templates for RT-PCR amplification. As shown in Fig. 7, products with an expected length of 430 bp (*LsAQP1*) and about 940 bp (*LsActin*) were present in all tested organs and tissues. Thus, the obtained results indicate that *LsAQP1* is undergoing transcription in many different tissues and organs.

**Fig. 8:**
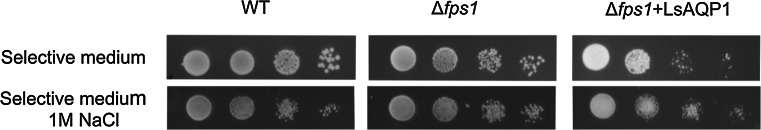
Analysis of LsAQP1 functionality. The growth complementation assay was performed for *S. cerevisiae* Δ*fps1* mutant cells depleted of functional Fps1 aquaglyceroporin and orthodox AQPs. The mutant cells were transformed with LsAQP1 encoding sequence and selected transformants were spotted in a 10× dilution series (1, 1:10, 1:100, 1:1000) on the solid selective media without NaCl (*upper panel*), and with 1 M NaCl (*lower panel*). The plates were incubated for 4 days at 28 °C. WT—isogenic wild type. The shown yeast colonies are typical results of three independent experiments

To investigate functionality of LsAQP1, its encoding sequence was used to transform Δ*fps1 S. cerevisiae* cells and the selected transformants were subjected to water transport analyses. It should be mentioned that Δ*fps1 S. cerevisiae* cells have neither functional aquaporins nor aquaglyceroporins. Thus, these mutant cells represent a good system for testing a putative aquaporins (Ahmadpour et al. 2013). When a functional water channel is expressed, the cells placed on a high osmolarity medium should display reduced growth or survival (Staniscuaski et al. 2013). As shown in Fig. 8, high osmolarity medium containing 1 M NaCl did not affect the growth of Δ*fps1* cells but clearly weakened the growth of the isogenic wild type cells (WT) containing the functional Fps1 classified as aquaglyceroporin. The growth of Δ*fps1* + LsAQP1 cells in the presence and absence of 1 M NaCl indicated that deficiency of Fps1 was at least partially complemented by LsAQP1. The Δ*fps1* + LsAQP1 cells displayed distinctly changed phenotype when compared to the mutant cells, and the phenotype resembled that of the isogenic wild type. For example, in the presence of NaCl, the growth of Δ*fps1* + LsAQP1 cells was delayed or the cell survival decreased when compared to the growth that occurred in the absence of NaCl (Fig. 8). The observation indicates the presence of functional LsAQP1 protein in the cells displaying activity similar to Fps1.

## Discussion

According to our knowledge, we have identified for the first time sequences encoding proteins that appear to be AQPs of three snail species (Mollusca; Gastropoda; Pulmonata; Lymnaeidae), i.e., *L. stagnalis, Catascopia occulta,* and *Stagnicola palustris.* The proteins, named LsAQP1, CoAQP1, and SpAQP1, respectively, are apparently the first described snail AQPs.

It is reasonable to assume that comparison of *LsAQP1, SpAQP1,* and *CoAQP1* nucleotide sequences could provide information that addresses the evolutionary relationships between studied lymnaeids. Our results confirmed that *L. stagnalis* and *S. palustris* are species more closely related in comparison to *C. occulta.* The obtained cDNA sequences encoding LsAQP1 and SpAQP1 are more similar to each other than to CoAQP1 (Fig. 1). However, the genetic distance between the studies species, as reflected by the number of nucleotide substitutions in *LsAQP1, CoAQP1*, and *SpAQP1* encoding sequences, does not appear at the level of amino acid sequences (Fig. 2). Due to synonymous changes in nucleotide sequences, the number of different amino acids in a given position is comparable for *L. stagnalis* with *S. palustris* and *L. stagnalis* with *C. occulta*. Thus, the phylogenetic tree that includes the amino acid sequences, shown in Fig. 6, supports the identification of LsAQP1, CoAQP1, and SpAQP1 as members of AQP1-like subfamily, but does not reveal relationships of encoding genes.

The analysis of amino acid sequences predicted for LsAQP1, SpAQP1, and CoAQP1, including a direct nucleotide and amino acid sequence analysis as well as hydropathy plots and 3D homology models, revealed the structural features characteristic for AQPs: two NPA motives and ar/R region. In addition, the ar/R region containing histidine residue that excludes the passage of larger solutes by steric occlusion, and a lack of extracellular vestibule (the typical structure of AQGPs) point their affiliation to orthodox AQPs, i.e., those permeating water molecules mainly (Figs. 2, 4, 5a–d). The similarity between LsAQP1, CoAQP1, SpAQP1, and vertebrate AQP4 representing orthodox AQPs was also confirmed by BLAST analysis (Fig. 3). In addition, as shown in Fig. 3, within the amino acid sequences of LsAQP1, CoAQP1, and SpAQP1 we found five amino acid residues described as the key positions (P1–P5) that could play an important role in the structure and function of orthodox AQPs (Froger et al. 1998). In the studied proteins, the P1 position was occupied by Gln101, P2 by Ser196, P3 by Ala200, P4 by Phe212, and P5 by Trp213. All these positions were, to a great extent, conservative for AQP transporting mainly water; only P1 is more frequently occupied by threonine than by glutamine (Froger et al. 1998). Due to the similar chemical nature of these two amino acids (coexisting in AQPs in the P1 position), we propose that this replacement may have not been significant for the functioning of the water channel. We also found that the ISGGH sequence localized before the first NPA motif in LsAQP1, CoAQP1, and SpAQP1 (Fig. 3) is the next feature conserved in orthodox AQPs. Consequently, our data suggest that LsAQP1, CoAQP1, and SpAQP1 are probably representatives of orthodox AQPs.

An amphipathic nature of the inside of the channel pore is a prerequisite for transferring water molecules. One hydrophobic and two hydrophilic regions form the pathway through the channel. In human AQP4, the hydrophobic region is formed by the side chains of Phe77, Ile81, Val85, Leu170, Ile174, and Val197 (Ho et al. 2009). LsAQP1, CoAQP1, and SpAQP1 posses the hydrophobic amino acids in the same positions as human AQP4 and three of them are identical. The differences concern Val in position 81, Tyr170, and Val174. Despite these three replacements, the hydrophobic nature of this region is maintained. A similar situation can be seen with the two hydrophilic regions formed by backbone carbonyls (Ho et al. 2009). In human AQP4, the first region is formed by Gly93, Gly94, His95, Ile96; the second region by Gly209, Ala210, Ser211, and Met212. In the case of the studied proteins, the first region was represented by Gly93, Gly94, His95, and Val96, whereas the second region consisted of Ala210, Ser211, Leu212, and Gly209 (in LsAQP1) or Arg209 (in SpAQP1 and CoAQP1). Thus, the presence of two hydrophilic and one hydrophobic regions within the putative channels formed by LsAQP1, SpAQP1, and CoAQP1 could provide conditions for water transport in two directions, just as it happens in other AQPs.

Within amino acid sequences of LsAQP1, CoAQP1, and SpAQP1, two putative phosphorylation sites occurred, namely Ser90, in the B loop and Thr247 in the C-terminal domain (Fig. 2). One of them, Ser90, was localized precisely in the same region as Ser111 in human AQP4. They can, therefore, be regarded as counterparts. Since it has been proven that phosphorylation of Ser111 contributes to the increase in the speed of water transport (Yukutake and Yasui 2010), we postulate that Ser90 in LsAQP1, CoAQP1, and SpAQP1 might be regarded as an element of the gating mechanism controlled by phosphorylation events (Gunnarson et al. 2005, 2008).

Despite the low levels of protein sequence identity between and within aquaporins and aquaglyceroporins, the published data indicate a high evolutionary conservation of all MIP representatives (Soto et al. 2012). The amino acid sequences of LsAQP1, CoAQP1, SpAQP1, and a few sequences of putative molluscan aquaporins found in GenBank were put on the Soto’s phylogenetic tree to determine to which subfamily (Soto et al. 2012) they might be classified (Fig. 6). The result shows that the cluster containing LsAQP1, CoAQP1, and SpAQP1 is a part of AQP1-like subfamily (Soto et al. 2012). The localization of LsAQP1, CoAQP1, and SpAQP1 on the phylogenetic tree seems to support the conclusion derived from nucleotide and amino acid sequence analyses that the proteins could function as orthodox AQPs.

The RT-PCR experiment showed the presence of *LsAQP1* mRNA in all tested tissues and organs (Fig. 7). Interestingly, similar results have been obtained recently for another putative AQP identified in dogfish *Squalus acanthias*, which is localized in the AQP4 cluster (Soto et al. 2012). *Squalus acanthias* *AQP4* expression studies revealed the presence of *AQP4* mRNA in each tissue examined (Cutler et al. 2012). In addition, human AQP4 is the widespread protein expressed in many different tissues and organs in the human body: the nervous system, the muscles, the stomach, and the kidney (Takata et al. 2004). Thus, the observed prevalence of *LsAQP1* transcript in the snail body is congruent with wide tissue distribution of members of AQP1-like subfamily clade; i.e., AQP4.

The yeast growth complementation assay revealed that *LsAQP1* codes for a protein displaying activity similar to Fps1, known also as *S. cerevisiae* aquaglyceroporin. Expression of LsAQP1 in Δ*fps1* cells affect the cell growth or survival making the cell phenotype similar to that observed for the isogenic wild type (Fig. 8). The influence of NaCl on AQP function and expression has been tested especially for mammalian brain and heart (Cao et al. 2012; Rutkovskiy et al. 2012). It has been observed that NaCl can serve as a factor able to regulate AQP function. Moreover, high NaCl concentration may cause both down- and up-regulation of AQP expression. It has been also observed that two orthodox AQP (PvAQP1 and PvAQP2) identified in larvae of the sleeping chironomid, *Polypedilum vanderplanki*, and tested in *Xenopus* oocytes, have different expression patterns after NaCl treatment (Kikawada et al. 2008). The results of the yeast growth complementation test suggest NaCl-regulated expression of *LsAQP1* and their contribution to water transport, but further studies concerning functional characterization of the encoded protein are still needed.

## Conclusions

In summary, the obtained results point at identification of new proteins termed LsAQP1, CoAQP1, and SpAQP1 that appear to be first aquaporins described in snails. The conclusion is supported by identification of AQP structural elements, observed phylogenetic relationships with other AQPs, and the results of the yeast growth complementation test. Moreover, detection of the transcript of *LsAQP1* gene in many different organs of *L. stagnalis* suggests important role of lymnaeid AQPs in water transport processes in various tissues, although further study concerning the functional characterization is indispensable to understand the physiological roles played by all new discovered gastropod AQPs.

## Electronic supplementary material

Below is the link to the electronic supplementary material.

Supplementary material 1 (DOC 5416 kb)

## References

[CR1] Agre P, King LS, Yasui M, Guggino WB, Ottersen OP, Fujiyoshi Y, Engel A, Nielsen S (2002). Aquaporin water channels—from atomic structure to clinical medicine. J Physiol.

[CR2] Ahmadpour D, Geijer C, Tamás MJ, Lindkvist-Petersson K, Hohmann S (2013) Yeast reveals unexpected roles and regulatory features of aquaporins and aquaglyceroporins. Biochim Biophys Acta. doi:10.1016/j.bbagen.2013.09.027 10.1016/j.bbagen.2013.09.027 24076236

[CR3] Altschul SF, Gish W, Miller W, Myers EW, Lipman DJ (1990). Basic local alignment search tool. J Mol Biol.

[CR4] Bargues MD, Vigo M, Horák P, Dvorak J, Patzner RA, Pointier JP, Jackiewicz M, Meier-Brook C, Mas-Coma S (2001). European Lymnaeidae (Mollusca: Gastropoda), intermediate hosts of trematodiases, based on nuclear ribosomal DNA ITS-2 sequences. Infect Genet Evol.

[CR5] Bargues MD, Horák P, Patzner RA, Pointier JP, Jackiewicz M, Meier-Brook C, Mas-Coma S (2003). Insight into relationships of Palearctic and Nearctic lymnaeids (Mollusca: Gastropoda) by rDNA ITS-2 sequencing and phylogeny of stagnicoline intermediate host species of Fasciola hepatica. Parasite.

[CR6] Beitz E, Wu B, Holm LM, Schultz JE, Zeuthen T (2006). Point mutations in the aromatic/arginine region in aquaporin1 allow passage of urea, glycerol, ammonia, and protons. Proc Natl Acad Sci USA.

[CR7] Bienert GP, Møller AL, Kristiansen KA, Schulz A, Møller IM, Schjoerring JK, Jahn TP (2007). Specific aquaporins facilitate the diffusion of hydrogen peroxide across membranes. J Biol Chem.

[CR8] Blom N, Gammeltoft S, Brunak S (1999). Sequence and structure-based prediction of eukaryotic protein phosphorylation sites. J Mol Biol.

[CR9] Blom N, Sicheritz-Pontén T, Gupta R, Gammeltoft S, Brunak S (2004). Prediction of post-translational glycosylation and phosphorylation of proteins from the amino acid sequence. Proteomics.

[CR10] Campbell EM, Ball A, Hoppler S, Bowman AS (2008). Invertebrate aquaporins: a review. J Comp Physiol B.

[CR11] Cao C, Yu X, Liao Z, Zhu N, Hou H, Wang M, Ji G, She H, Luo Z, Yue S (2012). Hypertonic saline reduces lipopolysaccharide-induced mouse brain edema through inhibiting aquaporin 4 expression. Crit Care.

[CR12] Cutler CP, Maciver B, Cramb G, Zeidel M (2012). Aquaporin 4 is a ubiquitously expressed isoform in the dogfish (*Squalus acanthias*) shark. Front Physiol.

[CR13] Endeward V, Musa-Aziz R, Cooper GJ, Chen LM, Pelletier MF, Virkki LV, Supuran CT, King LS, Boron WF, Gros G (2006). Evidence that aquaporin 1 is a major pathway for CO2 transport across the human erythrocyte membrane. FASEB J.

[CR14] Felsenstein J (1985). Confidence limits on phylogenies: an approach using the bootstrap. Evolution.

[CR15] Feng ZP, Zhang Z, van Kesteren RE, Straub VA, van Nierop P, Jin K, Nejatbakhsh N, Goldberg JI, Spencer GE, Yeoman MS, Wildering W, Coorssen JR, Croll RP, Buck LT, Syed NI, Smit AB (2009). Transcriptome analysis of the central nervous system of the mollusk *Lymnaea stagnalis*. BMC Genom.

[CR16] Froger A, Tallur B, Thomas D, Delamarche C (1998). Prediction of functional residues in water channels and related proteins. Protein Sci.

[CR17] Frohman MA (1993). Rapid amplification of cDNA ends: thermal RACE. Methods Enzymol.

[CR18] Gunnarson E, Axehult G, Baturina G, Zelenin S, Zelenina M, Aperia A (2005). Lead induces increased water permeability In astrocytes expressing aquaporin 4. Neuroscience.

[CR19] Gunnarson E, Zelenina M, Axehult G, Song Y, Bondar A, Krieger P, Brismar H, Zelenin S, Aperia A (2008). Identification of a molecular target for glutamate regulation of astrocyte water permeability. Glia.

[CR20] Gupta AB, Verma RK, Agarwal V, Vajpai M, Bansal V, Sankararamakrishnan R (2012). MIPModDB: a central resource for the superfamily of major intrinsic proteins. Nucleic Acids Res.

[CR21] Hall TA (1999). BioEdit: a user-friendly biological sequence alignment editor and analysis program for Windows 95/98/NT. Nucl Acids Symp Ser.

[CR22] Hasegawa H, Ma T, Skach W, Matthay MA, Verkman AS (1994). Molecular cloning of a mercurial-insensitive water channel expressed in selected water-transporting tissues. J Biol Chem.

[CR23] Herrera M, Garvin JL (2011). Aquaporins as gas channels. Pflugers Arch – Eur J Physiol.

[CR24] Herrera M, Hong NJ, Garvin JL (2006). Aquaporin-1 transports NO across cell membranes. Hypertension.

[CR25] Ho JD, Yeh R, Sandstrom A, Chorny I, Harries WE, Robbins RA, Miercke LJ, Stroud RM (2009). Crystal structure of human aquaporin 4 at 1.8 Ǻ and its mechanism of conductance. Proc Natl Acad Sci USA.

[CR26] Holm LM, Jahn TP, Møller AL, Schjoerring JK, Ferri D, Klaerke DA, Zeuthen T (2005). NH3 and NH4 + permeability in aquaporin-expressing Xenopus oocytes. Pflugers Arch.

[CR27] Ishibashi K (2006). Aquaporin subfamily with unusual NPA boxes. Biochim Biophys Acta.

[CR28] Ishibashi K, Hara S, Kondo S (2009). Aquaporin water channels in mammals. Clin Exp Nephrol.

[CR29] Jackiewicz M (1993). Phylogeny and relationships within European species of the family Lymnaeidae (Gastropoda: Pulmonata: Basommatophora). Folia Malacol.

[CR30] Jackiewicz M (1998). European species of the family Lymnaeidae (Gastropoda, Pulmonata, Basommatophora). Genus.

[CR31] Kataoka N, Miyake S, Azuma M (2009). Aquaporin and aquaglyceroporin in silkworms, differently expressed in the hindgut and midgut of Bombyx mori. Insect Mol Biol.

[CR32] Kaufmann N, Mathai JC, Hill WG, Dow JA, Zeidel ML, Brodsky JL (2005). Developmental expression and biophysical characterization of a *Drosophila melanogaster* aquaporin. Am J Physiol Cell Physiol.

[CR33] Kikawada T, Saito A, Kanamori Y, Fujita M, Śnigórska K, Watanabe M, Okuda T (2008). Dehydratation-inducible changes in expression of two aquaporins in the sleeping chironomid, *Polypedilum vanderplanki*. Biochim Biophys Acta.

[CR34] Kurowski MA, Bujnicki JM (2003). GeneSilico protein structure prediction meta-server. Nucleic Acids Res.

[CR35] Kyte J, Doolittle RF (1982). A simple method for displaying the hydropathic character of a protein. J Mol Biol.

[CR36] Ma JF, Tamai K, Yamaji N, Mitani N, Konishi S, Katsuhara M, Ishiguro M, Murata Y, Yano M (2006). A silicon transporter in rice. Nature.

[CR37] Master V (1993) Chicago. http://www.bio.net/hypermail//immuno/1993-September/000528.html

[CR38] Meier-Brook C, Bargues MD (2002). *Catascopia*, a new genus for three Nearctic and one Palaearctic stagnicoline species (Gastropoda: Lymnaeidae). Folia Malacol.

[CR39] Meng YL, Liu Z, Rosen BP (2004). As(III) and Sb(III) uptake by GlpF and efflux by ArsB in *Escherichia coli*. J Biol Chem.

[CR40] Preston GM, Carroll TP, Guggino WB, Agre P (1992). Appearance of water channels in Xenopus oocytes expressing red cell CHIP28 protein. Science.

[CR41] Rutkovskiy A, Mariero LH, Nygard S, Stenslokken KO, Valen G, Vaage J (2012). Transient hyperosmolality modulates expression of cardiac aquaporins. Biochem Biophys Res Commun.

[CR42] Rybska E, Pacak A, Szweykowska-Kulińska Z, Lesicki A (2008). RAPD markers as a tool for analysis of relationships among selected species of Lymnaeidae (Gastropoda: Pulmonata). Folia Malacol.

[CR43] Saitou N, Nei M (1987). The neighbor-joining method—a new method for reconstructing phylogenetic trees. Mol Biol Evol.

[CR44] Saparov SM, Liu K, Agre P, Pohl P (2007). Fast and selective ammonia transport by aquaporin-8. J Biol Chem.

[CR45] Soto G, Alleva K, Amodeo G, Muschietti J, Ayub ND (2012). New insight into evolution of aquaporins from flowering plants and vertebrates: orthologous identification and functional transfer is possible. Gene.

[CR46] Spring JH, Robichaux SR, Hamlin JA (2009). The role of aquaporins in excretion in insects. J Exp Biol.

[CR47] Staniscuaski F, Paluzzi JP, Real-Guerra R, Carlini CR, Orchard I (2013). Expression analysis and molecular characterization of aquaporins in *Rhodnius prolixus*. J Insect Physiol.

[CR48] Sui H, Han BG, Lee JK, Walian P, Jap BK (2001). Structural basis of water-specific transport through the AQP1 water channel. Nature.

[CR49] Takata K, Matsuzaki T, Tajika Y (2004). Aquaporins: water Chanel proteins of the cell membrane. Prog Histochem Cytochem.

[CR50] Tamura K, Dudley J, Nei M, Kumar S (2007). MEGA4: Molecular Evolutionary Genetics Analysis (MEGA) software version 4.0. Mol Biol Evol.

[CR51] Tanaka M, Wallace IS, Takano J, Roberts DM, Fujiwara T (2008). NIP6;1 is a boric acid channel for preferential transport of boron to growing shoot tissues in Arabidopsis. Plant Cell.

[CR52] Thompson JD, Higgins DG, Gibson TJ (1994). CLUSTAL W: improving the sensitivity of progressive multiple sequence alignment through sequence weighting, position specific gap penalties and weight matrix choice. Nucleic Acid Res.

[CR53] Tomkowiak E, Pienkowska JR (2010). The current knowledge of invertebrate aquaporin water channels with particular emphasis on insect AQPs. Adv Cell Biol.

[CR54] Verkman AS, Mitra AK (2000). Structure and function of aquaporin water channels. Am J Physiol Renal Physiol.

[CR55] Vinarski MV (2003). The systematic position of *Lymnaea vulnerata* (Küster, 1862) and *L. occulta* (Jackiewicz, 1959) (Mollusca: Gastropoda: Lymnaeidae). Zoosyst Ross.

[CR56] Vinarski MV (2012). The Lymnaeid genus Catascopia Meier-Brook et Bargues, 2002 (Mollusca: Gastropoda: Lymnaeidae), its synonymy and species composition. Invertebr Zool.

[CR57] Vinarski MV, Glöer P (2008). Taxonomic notes on Euro-Siberian freshwater mollusks. 3. Galba occulta Jackiewicz, 1959 is a junior synonym of *Limnaea palustris* var. *terebra* Westerlund, 1885. Mollusca (Dresden).

[CR58] Wallner B, Elofsson A (2005). Identification of correct regions in protein models using structural, alignment and consensus information. Protein Sci.

[CR59] Welter-Schultes FW (2012). European non-marine molluscs, a guide for species identification.

[CR60] Yukutake Y, Yasui M (2010). Review: regulation of water permeability through aquaporin-4. Neuroscience.

[CR61] Zhang G, Fang X, Guo X, Li L, Luo R, Xu F, Yang P, Zhang L, Wang X, Qi H, Xiong Z, Que H, Xie Y, Holland PW, Paps J, Zhu Y, Wu F, Chen Y, Wang J, Peng C, Meng J, Yang L, Liu J, Wen B, Zhang N, Huang Z, Zhu Q, Feng Y, Mount A, Hedgecock D, Xu Z, Liu Y, Domazet-Lošo T, Du Y, Sun X, Zhang S, Liu B, Cheng P, Jiang X, Li J, Fan D, Wang W, Fu W, Wang T, Wang B, Zhang J, Peng Z, Li Y, Li N, Wang J, Chen M, He Y, Tan F, Song X, Zheng Q, Huang R, Yang H, Du X, Chen L, Yang M, Gaffney PM, Wang S, Luo L, She Z, Ming Y, Huang W, Zhang S, Huang B, Zhang Y, Qu T, Ni P, Miao G, Wang J, Wang Q, Steinberg CE, Wang H, Li N, Qian L, Zhang G, Li Y, Yang H, Liu X, Wang J, Yin Y, Wang J (2012). The oyster genome reveals stress adaptation and complexity of shell formation. Nature.

