# Supplementary material for: Molecular Identification of First Putative Aquaporins in Snails
Source: J Membr Biol. 2014 Jan 21;247(3):239–52. doi: 10.1007/s00232-014-9629-0 (PMC3930841; doi:10.1007/s00232-014-9629-0)
Supplement: Supplementary file 1 — Supplementary material 1 (DOC 5416 kb) [file 232_2014_9629_MOESM1_ESM.doc]

a b


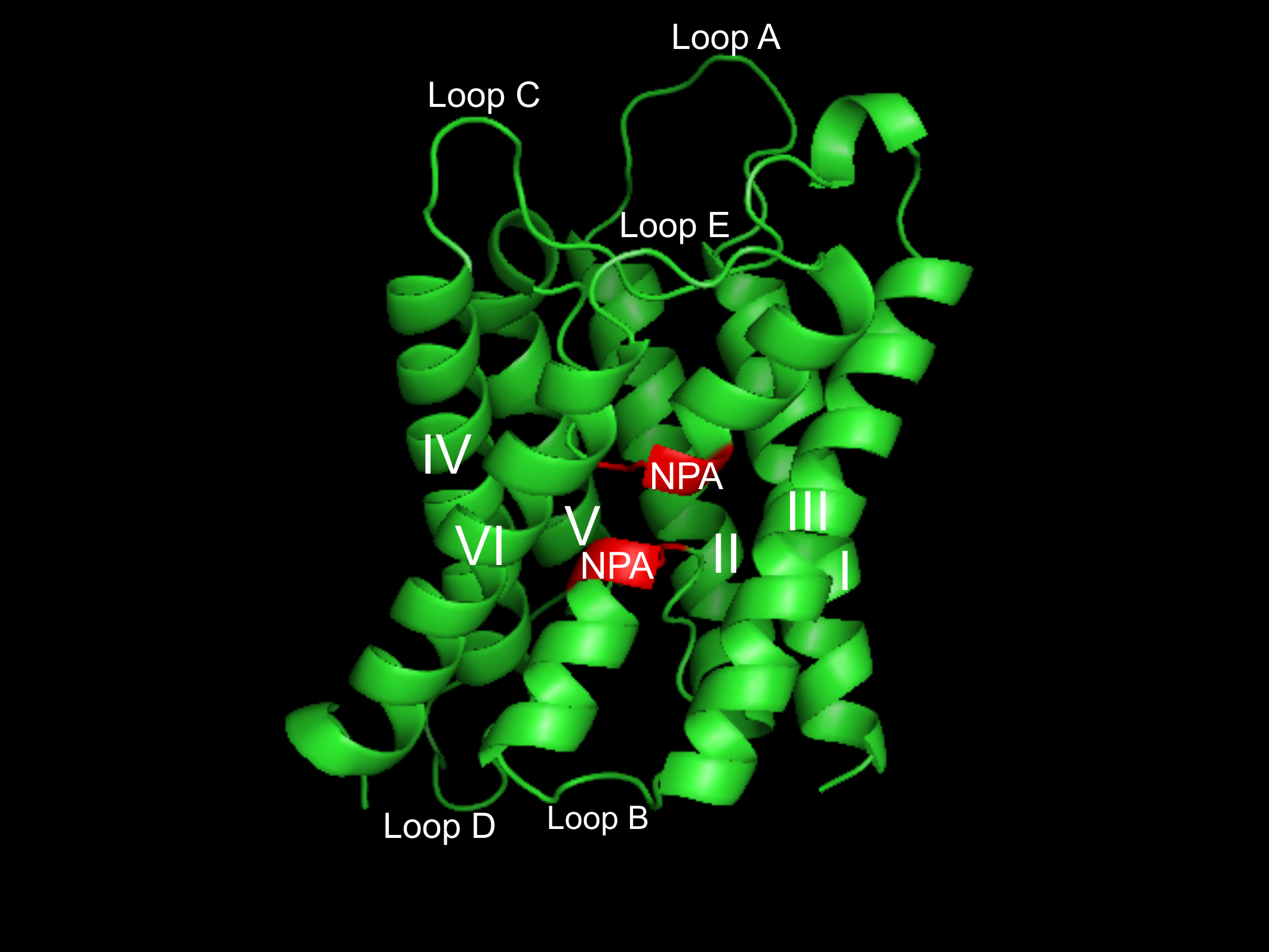

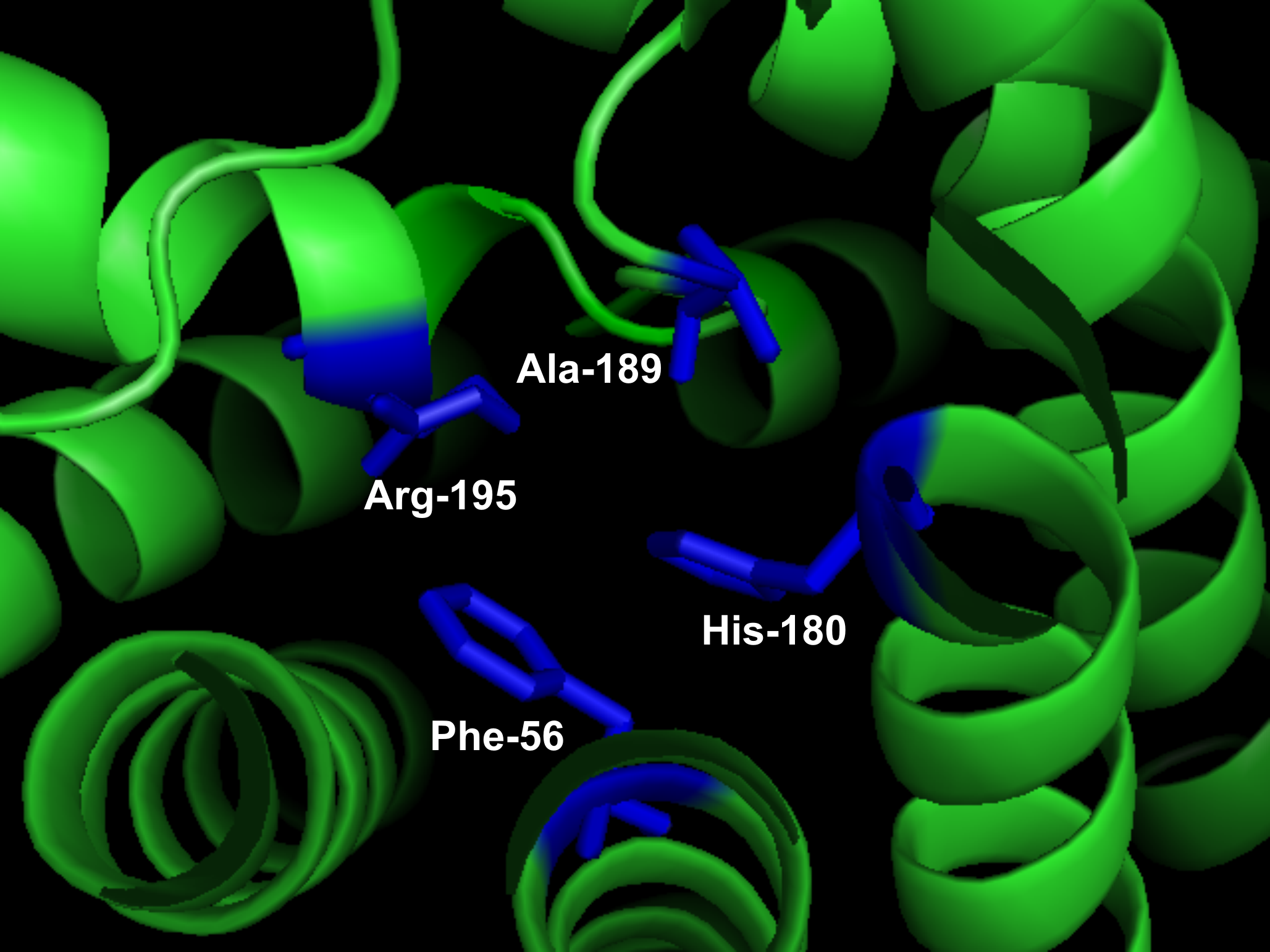


c d


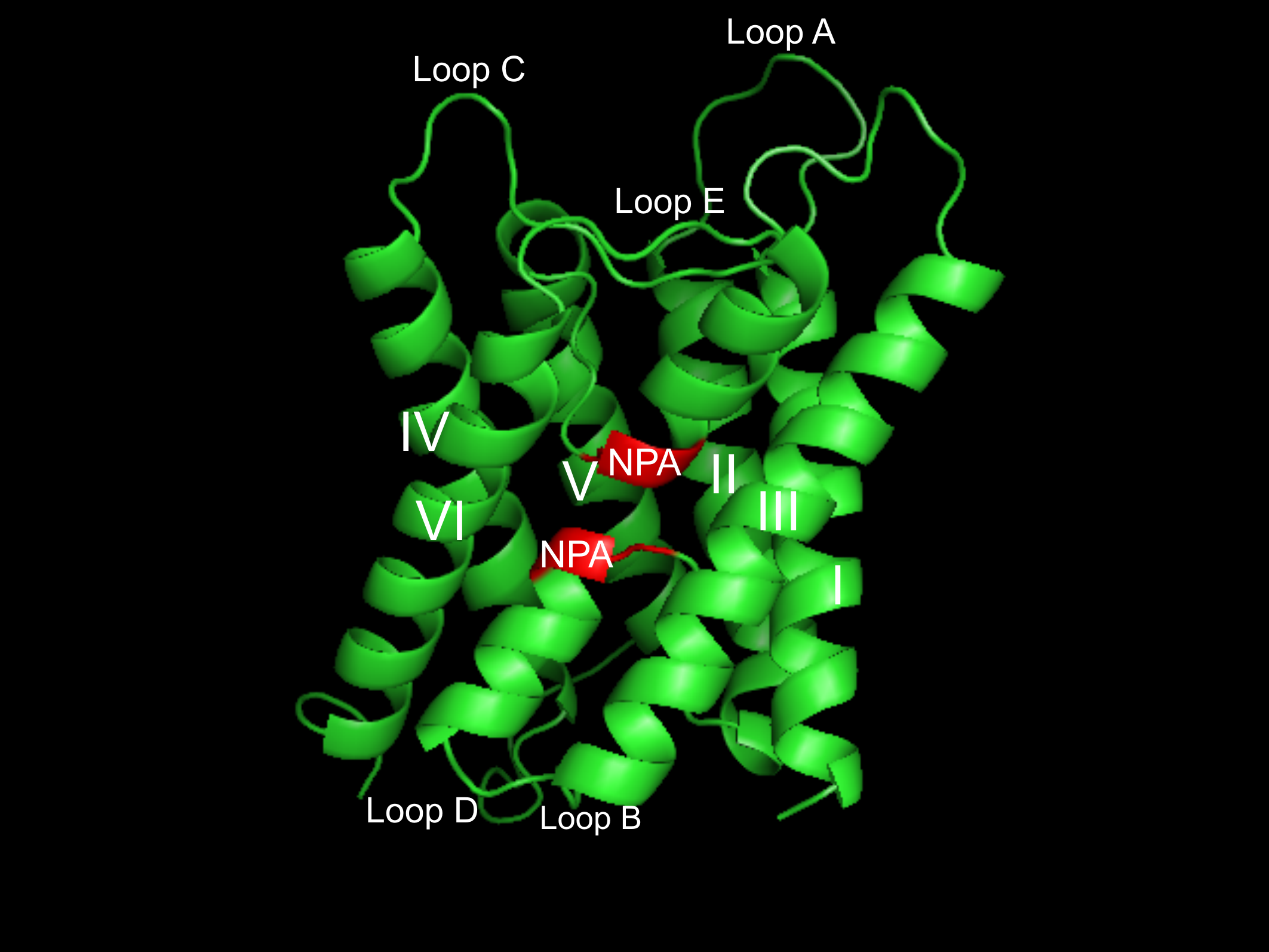

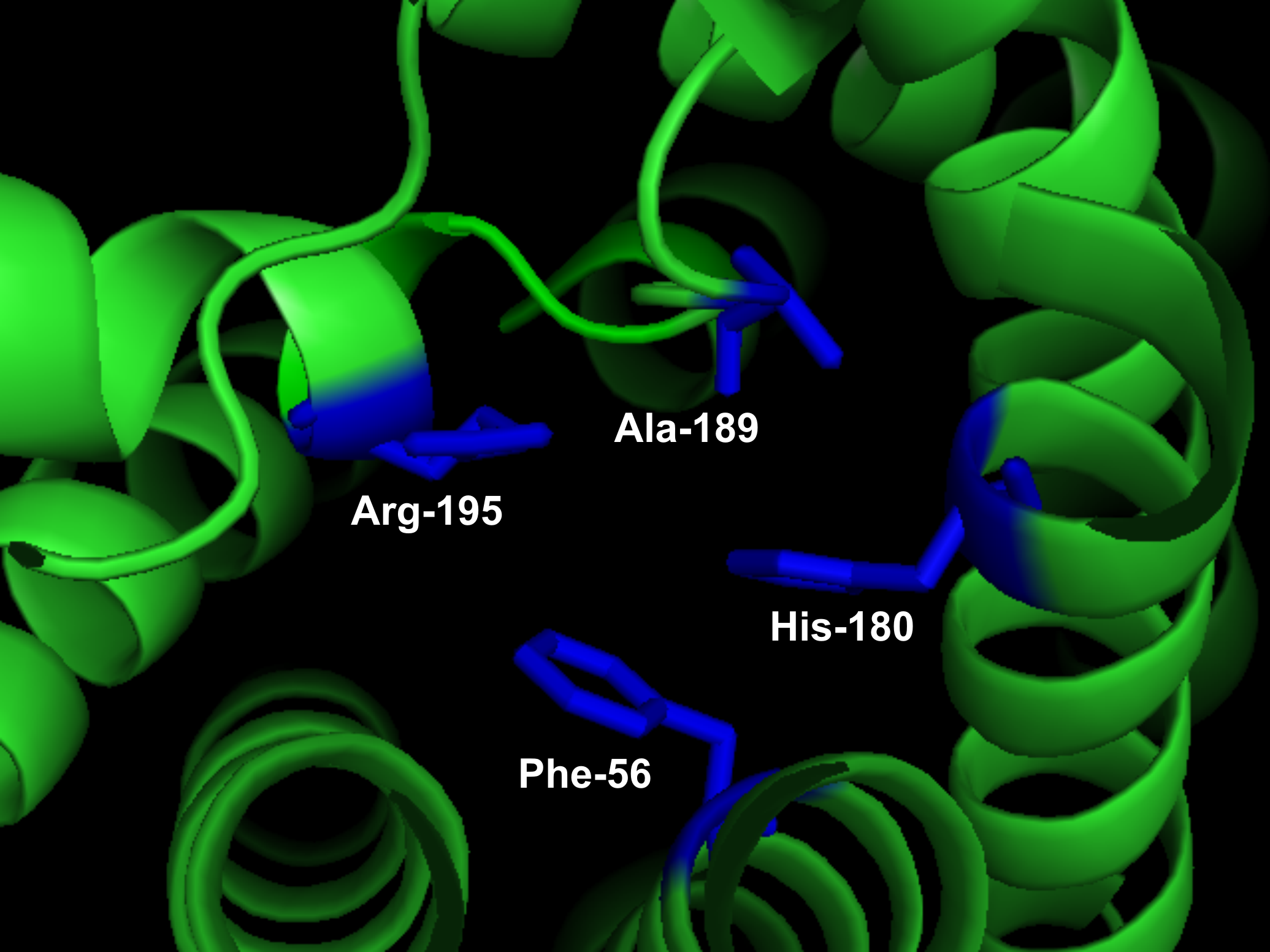


**Sup. 1** Three-dimensional structure homology model of CoAQP1 and SpAQP1. The crystal structure of *Homo sapiens* AQP4 (PDB ID: 3GD8) was applied as a template. **a** Three-dimensional homology model of CoAQP1 presented parallel to the plane of the membrane. Two NPA motives are shown in red. The transmembrane domains and loops are indicated by numbers and letters, respectively. **a** Amino acid residues building the putative selectivity filter known also as ar/R region (Phe56, His180, Ala189, and Arg195) of CoAQP1 are presented in blue. **c** Three-dimensional structure of human SpAQP1 presented in the same orientation as CoAQP1. Description of the picture is analogous to the above. **d** The structure of SpAQP1 selectivity filter with amino acids building this region shown in blue
